# Bidirectional crosstalk between epithelial–mesenchymal plasticity and IFN*γ*-induced PD-L1 expression promotes tumour progression

**DOI:** 10.1098/rsos.220186

**Published:** 2022-11-02

**Authors:** Gerhard A. Burger, Daphne N. Nesenberend, Carlijn M. Lems, Sander C. Hille, Joost B. Beltman

**Affiliations:** ^1^ Division of Drug Discovery and Safety, Leiden University, Leiden, The Netherlands; ^2^ Mathematical Institute, Leiden Academic Centre for Drug Research, Leiden University, Leiden, The Netherlands

**Keywords:** epithelial-mesenchymal transition (EMT), immune evasion, PD-L1, interferon gamma

## Abstract

Epithelial–mesenchymal transition (EMT) and immunoevasion through upregulation of programmed death-ligand 1 (PD-L1) are important drivers of cancer progression. While EMT has been proposed to facilitate PD-L1-mediated immunosuppression, molecular mechanisms of their interaction remain obscure. Here, we provide insight into these mechanisms by proposing a mathematical model that describes the crosstalk between EMT and interferon gamma (IFN*γ*)-induced PD-L1 expression. Our model shows that via interaction with microRNA-200 (miR-200), the multi-stability of the EMT regulatory circuit is mirrored in PD-L1 levels, which are further amplified by IFN*γ* stimulation. This IFN*γ*-mediated effect is most prominent for cells in a fully mesenchymal state and less strong for those in an epithelial or partially mesenchymal state. In addition, bidirectional crosstalk between miR-200 and PD-L1 implies that IFN*γ* stimulation allows cells to undergo EMT for lower amounts of inducing signal, and the presence of IFN*γ* accelerates EMT and decelerates mesenchymal–epithelial transition (MET). Overall, our model agrees with published findings and provides insight into possible mechanisms behind EMT-mediated immune evasion, and primary, adaptive, or acquired resistance to immunotherapy. Our model can be used as a starting point to explore additional crosstalk mechanisms, as an improved understanding of these mechanisms is indispensable for developing better diagnostic and therapeutic options for cancer patients.

## 1. Introduction

Activating invasion and metastasis and evading immune destruction are well-established hallmarks of cancer, complementary capabilities that enable tumour growth and metastatic dissemination [1]. Because metastasis is the main cause of cancer mortality, a thorough understanding of the interaction between these hallmarks is essential for developing therapeutic approaches, yet they are often studied separately [2]. Consequently, the interplay between metastatic dissemination and immune evasion remains poorly understood.

In recent years, epithelial–mesenchymal plasticity (EMP), the ability of cells to interconvert between intermediate E/M phenotypes along the epithelial–mesenchymal transition (EMT) spectrum [3], has been extensively studied because of its crucial role in invasion and metastasis (reviewed in [4–6]). Moreover, EMP is associated with therapeutic resistance (reviewed in [5,7]), including resistance to immunotherapy (reviewed in [8]). One of the mechanisms through which cancer cells acquire immune resistance is by activation of immune checkpoint pathways, which under physiological conditions are indispensable for self-tolerance and modulation of the immune response [9].

A frequently upregulated checkpoint protein in tumours is programmed death-ligand 1 (PD-L1) [10]. Its corresponding receptor programmed death 1 (PD-1) is expressed on the cell membrane of T cells, and PD-1–PD-L1 interaction induces a variety of immunosuppressive effects, such as inhibition of T cell proliferation, survival and effector functions [11]. Tumour cells can express PD-L1 by two general mechanisms: adaptive immune resistance, where PD-L1 expression is upregulated in response to inflammatory factors (such as interferon gamma (IFN*γ*)) produced by an anti-tumour immune response, and innate immune resistance, where PD-L1 expression is upregulated in response to constitutive oncogenic signalling [9]. Such constitutive signalling could, for example, be caused by EMT, and indeed several links between EMT and PD-L1-mediated immunoevasion have been reported in the literature (reviewed by Jiang and Zhan [12], who conclude that additional mechanistic studies are urgently needed).

Here, we study the post-transcriptional regulation of PD-L1 by the microRNA-200 (miR-200)–zinc finger E-box-binding homeobox 1 (ZEB1) axis, which is one of the proposed mechanisms underlying the interplay between EMT and immune resistance [13–15]. The binding of miR-200 to the mRNA of PD-L1 inhibits mRNA translation and in general such binding can stimulate miRNA decay [16,17]. The miR-200–ZEB1 axis is ‘a motor of cellular plasticity’ [18] and is considered to be part of the EMT core regulatory network [19]. Various mathematical models of EMT that include the miR-200–ZEB1 axis have been developed, which have contributed to a better mechanistic understanding of EMT (reviewed in [3,20,21]). Recently, Sahoo *et al.* [22] presented a mathematical model interconnecting a minimal EMT network and PD-L1 using shifted Hill functions to investigate immune-evasive strategies of hybrid E/M states, assuming an indirect effect of PD-L1 on EMT. Model analysis showed that both the stable hybrid and full EMT phenotypes resulting from the model were associated with high PD-L1 levels. However, although EMT scores determined from gene expression levels across a large panel of cell lines indeed exhibited a pattern of gradually increasing PD-L1 expression with the increasing EMT score, there was no clear dichotomy as expected from the mathematical model. This difference may be explained by the indirect nature of the feedback of PD-L1 to EMT as implemented in the model of Sahoo *et al.* [22]. Moreover, the impact of adaptive, IFN*γ*-driven PD-L1 expression, i.e. the primary reason for PD-L1 upregulation [23,24], was not taken into account in their model.

To describe the full interactions expected between adaptive and innate immune resistance and to study the impact of direct, mutual feedback between EMT and PD-L1, we connected a ‘core’ EMT model to a model for IFN*γ*-induced PD-L1 expression, which we developed based on an extension of a published JAK–STAT model [25]. For EMT, we used the ternary chimera switch (TCS) model by Jolly *et al.* [26], which is a simplification compared with the prior work [27]. Combining these two models was achieved by adding a mutually inhibitory feedback loop between miR-200 and PD-L1, which we described using appropriate miRNA–mRNA dynamics [28]. The analysis of our model shows that IFN*γ*-induced PD-L1 expression is expected to greatly accelerate EMT and decelerate the reverse mesenchymal–epithelial transition (MET) process. Moreover, IFN*γ*-induced PD-L1 lowers the required level of EMT-inducing signal, leading to an overall larger probability of EMT in tumours with a high IFN*γ* expression compared with tumours with a low expression. Vice versa, and consistent with the study by Sahoo *et al.* [22], a full EMT induced via other signals greatly upregulates PD-L1 expression, which IFN*γ* further amplifies. However, in our model, the hybrid EMT phenotype only moderately affects PD-L1 expression, which is due to the mutual feedback between EMT and PD-L1. Finally, we show that our model findings are broadly consistent with published experimental results in an extensive comparison with experimental data. Overall, our analysis illustrates how crosstalk between EMP and IFN*γ*-induced PD-L1 production can result in immune evasion and contribute to invasion and metastasis.

## 2. Results

### 2.1. Modelling IFN*γ*-induced PD-L1 expression and EMT

**Figure 1.**
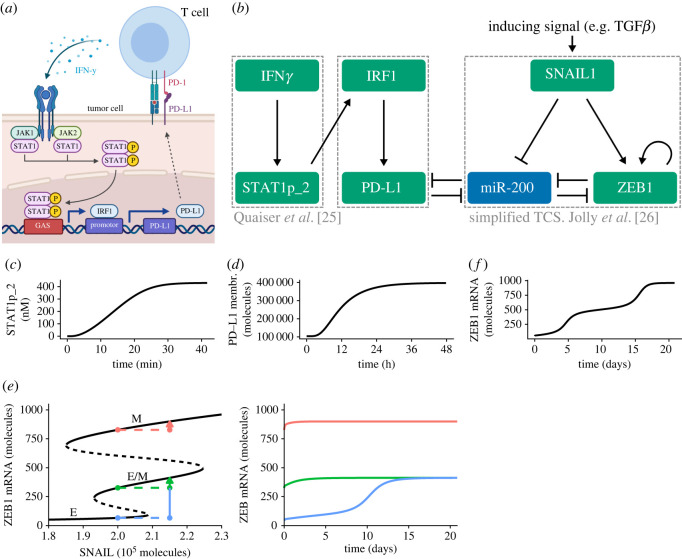
PD-L1 and EMT dynamics occur on distinct time scales. (*a*) Schematic diagram of the signalling pathway regulating IFN*γ*-induced PD-L1 expression. (*b*) Schematic diagram of the full model combining IFN*γ*-induced STAT signalling (left box), PD-L1 expression (middle box) and the simplified TCS model for EMT dynamics (right box). (*c*)–(*f*) Temporal dynamics of separate models (boxes in (*b*)) without connection to the other parts. This includes IFN*γ*-induced STAT1 signalling (*c*) with IFN*γ* = 0.1 nM, STAT1-induced PD-L1 expression (*d*) with STAT1p_2 = 430 nM and SNAIL1-induced EMT (*f*) with SNAIL1 = 2.3 × 10^5^ molecules. The bifurcation diagram in (*e*) illustrates how EMT phenotype depends on the inducing signal in the TCS model (left, black lines). The eventual phenotype also depends on the initial state of the system, illustrated by temporal dynamics after a sudden increase in SNAIL1 from 2.0 × 10^5^ molecules to 2.15 × 10^5^ molecules given three different initial states (coloured lines).

To study the interplay between IFN*γ*-induced PD-L1 expression and EMT, we first need a quantitative description of these major processes. Although interferon gamma (IFN*γ*)-induced PD-L1 expression via the JAK–STAT pathway (figure 1*a*) has been extensively studied [29,30], to the best of our knowledge, no mathematical model of this entire process exists. Existing models focus on IFN*γ*-induced JAK–STAT signalling (reviewed in [31]) and usually do not take into account direct targets of STAT such as interferon regulatory factors (IRFs) and interferon-stimulated genes. To model IFN*γ*-induced PD-L1 expression, we used the simplified JAK–STAT model by Quaiser and Mönnigmann [32] (figure 1*b*, left box), which is a truncated version of a more detailed model by Yamada *et al.* [33]. In this model, phosphorylated signal transducer and activator of transcription 1 (STAT1) homodimer (STAT1p_2) reaches its maximum at about 30 min after IFN*γ* exposure (figure 1*c*), in agreement with Brysha *et al.* [34]. This time scale is much shorter than the time scale of hours at which PD-L1 dynamics occur (figure 1*d*), in agreement with Kleijn *et al.* [35] and Ghosh *et al.* [36]. Therefore, we simplified this model further by describing the steady state of its output, i.e. STAT1p_2, with a Gompertz function (electronic supplementary material, Methods). We then extended this model with the production of IRF1 mediated by STAT1, and production of PD-L1 mediated by IRF1 (figure 1*b*, middle box, and electronic supplementary material, figure S1A). We modelled both of these processes with shifted Hill functions, which led to realistic IRF1 [37] and PD-L1 [36] dynamics (electronic supplementary material, figure S1B).

For EMT, various mathematical models have been developed (reviewed in [20,21]). Two well-known models of the miR34-SNAIL1 and miR200-ZEB1 axes, i.e. the core regulatory machinery of EMT [19], are the cascading bistable switches model (Tian *et al.* [38]; later revised by Zhang *et al.* [39]) and the TCS model [27]. We elected to use the simplified TCS model developed by Jolly *et al.* [26] (figure 1*b*, right box). Notably, the TCS model exhibits three stable states, representing an epithelial phenotype (E), an intermediate phenotype (E/M) and a mesenchymal phenotype (M) (figure 1*e*, left, black lines). Consistent with the results for the full TCS model (cf. fig. 6 in [27]), a full EMT in the simplified TCS model takes approximately 20 days, with a slowing down of the transitioning to the mesenchymal phenotype around ZEB1 expression levels for the hybrid phenotype (figure 1*f*). Both the initial state of the system and the EMT-inducing signal determine the phenotype that the system attains in the long run (figure 1*e*, coloured lines). Moreover, it is important to note the difference in time scales for the dynamics of the different model components: although signal transducer and activator of transcription (STAT) signalling and PD-L1 expression occur on a time scale of minutes and hours, a full EMT transition from E to M occurs on a time scale of days (figure 1*c*–*f*).

### 2.2. IFN*γ* amplifies the increase in PD-L1 caused by EMT primarily for mesenchymal cells

Because we aimed to study the impact of IFN*γ*-mediated PD-L1 on EMT and vice versa, we connected the separate model parts (figure 1*b*, boxes) by adding mutual inhibition between PD-L1 and miR-200 [13,14,40] using the theoretical framework for miRNA-transcription factor (TF) dynamics by Lu *et al.* [28]. This results in direct inhibition of PD-L1 by miR-200, as miR-200 binds to the mRNA of PD-L1 [13]. However, there is automatically also an inhibitory effect of PD-L1 on miR-200 because the modelled miRNA–mRNA interaction leads to additional miR-200 degradation (Lu *et al.* [28], see electronic supplementary material, Methods for details). This PD-L1 inhibitory effect on miR-200 also affects the miR-200 level in the absence of IFN*γ* because in the coupled model PD-L1 has a non-zero expression level (driven by EMT status). The basal miR-200 degradation rate in the TCS model should contain the influence of degradation by any molecule not described explicitly in that model, including PD-L1. Thus, to aim for a fair comparison between these structurally different models (i.e. TCS and our combined model (figure 1*b*)), we decreased the basal miR-200 degradation rate in the combined model until it approximately matched the bifurcation diagram for the TCS model (electronic supplementary material, figure S2).

**Figure 2.**
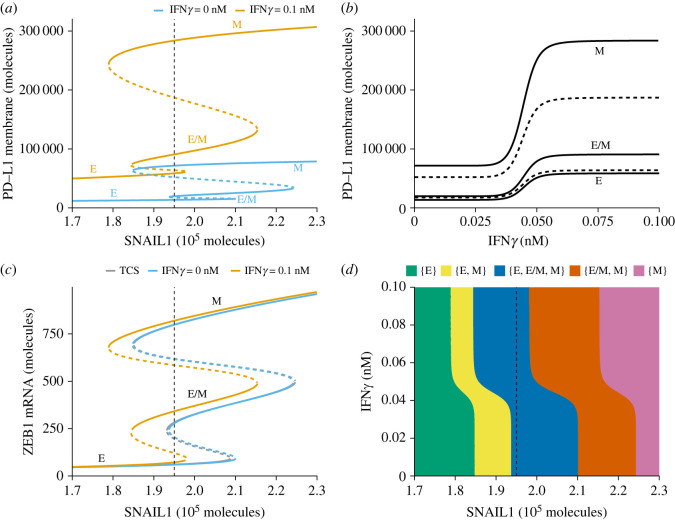
Model-predicted mutual influence of IFN*γ*-driven PD-L1 expression and EMT. (*a*–*c*) Bifurcation (*a*,*c*) and continuation (*b*) diagrams illustrating how the steady-state expression of PD-L1 on the membrane depends on SNAIL1 in the absence (blue) and presence (orange) of IFN*γ* (*a*), how the steady-state expression of PD-L1 on the membrane depends on IFN*γ*, considering a fixed SNAIL1 level of 1.95 × 10^5^ molecules (*b*) and how the steady state of ZEB1 mRNA depends on SNAIL1 in the TCS model (grey), and in our extended model in the absence (blue) or presence (orange) of IFN*γ* (*c*). Stable equilibria (representing E, E/M and M phenotypes) are indicated by solid lines and unstable equilibria by dashed lines. (*d*) Phase diagram showing how the presence of stable equilibria (coloured regions, indicated in the legend) depends on IFN*γ* and SNAIL1. Vertical dashed lines in (*a*), (*c*) and (*d*) show the SNAIL1 concentration used in (*b*).

Next, we studied how the system responds to different levels of SNAIL1 (activated via, for example, transforming growth factor beta (TGF*β*)) and IFN*γ* (figure 2). In the absence of IFN*γ*, the previously reported tristable ZEB1 levels [27] translate to similar tristability in PD-L1 expression at the cell membrane (blue line in figure 2*a*). Here, the highest PD-L1 expression occurs for mesenchymal cells and the lowest for epithelial cells. Note that the PD-L1 expression of hybrid cells is only slightly increased compared with epithelial cells, a prediction that is different from the recent study by Sahoo *et al.* [22], who predict an almost equal level of PD-L1 for the hybrid and mesenchymal phenotype. Notably, the mutual inhibition of miR-200 and PD-L1 did not result in additional bifurcation points. However, the combined model predicts that exposure of cells to IFN*γ* greatly amplifies PD-L1 expression for all phenotypes, thereby also amplifying the differences in PD-L1 expression between those EMT phenotypes (figure 2*a*, orange line; figure 2*b*). Nevertheless, in the presence of IFN*γ*, the hybrid phenotype has a PD-L1 expression level similar to that of epithelial cells, and only mesenchymal cells are expected to have substantially higher PD-L1 levels.

### 2.3. IFN*γ* promotes the occurrence of EMT

We next asked what the impact of IFN*γ* is on ZEB1, i.e. a central EMT-TF. In the absence of IFN*γ*, cells in our model undergo EMT for similar levels of SNAIL1 compared with the original TCS model (blue curve in figure 2*c* compared with the grey curve). Small differences between our model and the TCS model are caused by the bidirectional influence between PD-L1 and miR-200. In the presence of IFN*γ*, this mutual influence causes the bifurcation diagram to shift to the left (figure 2*c*, orange curve), because the expression of PD-L1 leads to a reduced amount of miR-200, in turn affecting EMT. This implies that according to our model IFN*γ* promotes EMT because both partial and full EMT more readily occur than in the absence of IFN*γ*.

We created a phase diagram to provide an overview of how the stability of EMT phenotypes depend on IFN*γ* and SNAIL1 levels (figure 2*d*). In this phase diagram, the IFN*γ*-induced leftward shift is clearly visible. In addition, it shows that the total range of SNAIL1 for which the hybrid E/M phenotype, a particularly aggressive EMT phenotype (Lüönd *et al.* [41], and reviewed in Jolly *et al.* [42]), can (co-)exist is unaffected. As the different EMT phenotypes express different levels of PD-L1 we additionally quantified these levels for each phenotype and for the average PD-L1 expression per stability region (electronic supplementary material, figure S3).

To investigate the role of the included JAK–STAT regulation, we created a simplified crosstalk model where we replaced JAK–STAT signalling with a generic inducing signal *I* (electronic supplementary material, figure S4A). The influence of this inducing signal is more gradual than the influence of IFN*γ* in the full model, yet the same leftward shift is present (electronic supplementary material, figure S4B, compared with figure 2*b*), suggesting that IFN*γ*-induced JAK–STAT signalling makes the response highly ‘switch-like’. In addition, we used a sensitivity analysis on parameters used in the negative-feedback loop between miR-200 and PD-L1, which showed that both the predicted leftward shift of SNAIL1 levels and the amplification of PD-L1 by IFN*γ* are robust to parameter change (electronic supplementary material, figure S5). In conclusion, our model predicts that local presence of IFN*γ* promotes partial and full EMT by lowering the threshold for additional EMT-inducing signals to induce (partial) EMT.

### 2.4. IFN*γ*-driven PD-L1 expression accelerates EMT

Besides the steady states for EMT status and PD-L1 expression that the combined regulatory network can reach in the long run, in practice, it will also matter how long it takes to reach these states. Therefore, we also investigated our combined regulatory network’s temporal dynamics and compared them with the temporal dynamics of the separate models.

**Figure 3.**
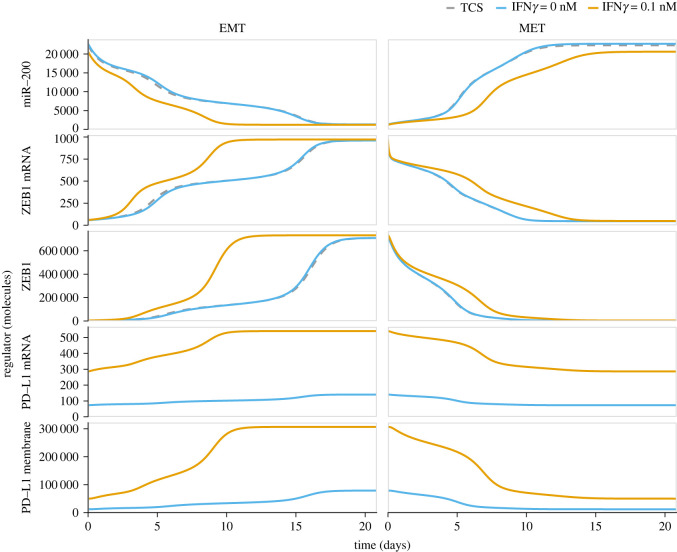
Temporal dynamics of EMP. EMT (left) and MET (right) for the simplified TCS model (dashed grey), and our combined model (figure 1*b*) with IFN*γ* = 0 nM (blue) and IFN*γ* = 0.1 nM (orange). For EMT, cells in an epithelial state with SNAIL1 = 1.7 × 10^5^ molecules undergo a full EMT by increasing SNAIL1 to 2.3 × 10^5^ molecules; for MET, SNAIL1 is decreased to the original value of 1.7 × 10^5^ molecules again.

To study the impact of PD-L1 expression on the temporal dynamics of an EMT transition and vice versa, we started with a stable epithelial phenotype (SNAIL1 = 1.7 × 10^5^ molecules) and simulated transition to a fully mesenchymal phenotype by a sudden increase of SNAIL1 (to 2.3 × 10^5^ molecules). In the absence of IFN*γ*, there is only a minor change in the temporal dynamics of EMT in our combined model relative to the simplified TCS model (blue versus grey lines in figure 3). However, as a consequence of the double-negative feedback loop between miR-200 and PD-L1, the decrease in miR-200 during EMT affects the PD-L1 mRNA and membrane PD-L1 (which are not present in the TCS model) at a similar time scale as miR-200. Thus, the effect of EMT on PD-L1 expression takes place on a much slower time scale than for PD-L1 expression driven by IFN*γ* (for comparison, see figure 1*d*).

In the presence IFN*γ*, the increased level of PD-L1 over time speeds up the miR-200 decrease (compared with the TCS model that does not consider an interaction with PD-L1). This is due to fast PD-L1-driven miR-200 degradation during the initial phase of EMT (electronic supplementary material, figure S6). Because of miR-200’s role as a critical suppressor of several EMT-TFs [43,44], this accelerates the EMT process by about 10 days, mainly by reducing the time cells remain in a state close to the hybrid (E/M) phenotype before converting to a fully mesenchymal phenotype (orange line in figure 3, left). Again, the PD-L1 expression level evolves slowly compared with the rapid increase observed on a time scale of hours for the model describing just IFN*γ* signalling (figure 1*d*). This difference in time scales of initial IFN*γ*-driven PD-L1 increase and of further PD-L1 increase due to EMT is especially apparent when considering a scenario where cells are simultaneously exposed to IFN*γ* and a TGF*β* (electronic supplementary material, figure S7, left column).

In summary, the double-negative feedback loop between miR-200 and PD-L1 accelerates EMT in the presence of IFN*γ*.

### 2.5. IFN*γ*-driven PD-L1 expression decelerates MET

Finally, we investigated whether similar changes occur in the temporal dynamics of the reverse process in which mesenchymal cells transition to an epithelial phenotype, i.e. MET. For this purpose, we started with a stable mesenchymal phenotype, where SNAIL1 = 2.3 × 10^5^ molecules, which we instantaneously decreased to 1.7 × 10^5^ molecules. In the simplified TCS model, this SNAIL1 decrease led to a direct transition from the mesenchymal into the epithelial phenotype (grey dashed line in figure 3, right), i.e. without a substantial slowing down of the dynamics around a hybrid phenotype.

As anticipated from the observed EMT dynamics showing that the double-negative feedback loop between PD-L1 and miR-200 favours the mesenchymal phenotype, this loop also affects MET, albeit in the opposite direction, i.e. it decelerates MET. This is primarily due to the long-lasting suppression of miR-200 (orange lines in figure 3, right). However, this effect is considerably smaller than the effect in the forward transition, which is probably the result of MET not transitioning through the hybrid state at a slow pace since the PD-L1–miR-200 interaction primarily accelerated EMT by shortening the time spent there (figure 3, left). Note that simultaneous lowering of SNAIL1 and removal of IFN*γ* do lead to an MET occurring at a similar speed as in the original TCS model (electronic supplementary material, figure S7, right column), demonstrating the important role of IFN*γ* in EMP.

In conclusion, the PD-L1–miR-200 double-negative feedback loop is expected to slow down MET considerably in the presence of IFN*γ*.

## 3. Discussion and conclusion

Here, we have used a mathematical model to describe the crosstalk between IFN*γ*-induced PD-L1 expression and EMT. We showed that merely adding the reported interaction between PD-L1 and miR-200 gives rise to tristability in the PD-L1 levels, where a mesenchymal state corresponds with high PD-L1 expression and an epithelial state with low PD-L1 expression. The difference in PD-L1 levels between the stable EMT states is amplified by adding IFN*γ* stimulation. In addition, we showed that adding this crosstalk reduces the amount of SNAIL1 required to undergo EMT in the presence of IFN*γ*. Finally, we showed that in the presence of IFN*γ*, this crosstalk accelerates the forward EMT process and decelerates the reverse MET process.

To assess the extent to which the model results agree with recent studies, we have compiled a summary of published experimental reports on the link between EMT and PD-L1 and indicated which findings are qualitatively consistent with our results (table 1). Note that we omitted several papers that propose different mechanisms underlying EMT/PD-L1 crosstalk and lacked experimental findings that could be compared with our simulations [54–57]. Overall, the simulated results of our model are in good agreement with the reported experimental findings.

**Table 1.** Summary of experimental reports regarding the crosstalk between EMT and PD-L1 and their consistency with our ODE model.

cancer type	description	match
—	association between EMT markers and PD-L1 expression in different cancer types [12]	yes
breast cancer	Induction of EMT in human breast cells using TGF*β* upregulated PD-L1 expression in a manner that was dependent on the activation of the PI3K/AKT and MEK/ERK pathways. In addition, manipulation of PD-L1 modulated the EMT status of breast cancer cells. Downregulation of PD-L1 using specific shRNA in mesenchymal-like cells led to partial EMT reversal. Conversely, overexpression of PD-L1 led to EMT [40].	yes^a^
	In an EMT-activated and a mesenchymal-like breast cancer cell line, upregulation of PD-L1 depended on SNAIL and ZEB1. In another EMT-activated cell line, silencing of ZEB1 (but not SNAIL) and overexpression of miR-200 family members strongly decreased PD-L1 expression. However, the treatment of EMT-activated breast cancer cells with TGF*β* did not affect PD-L1 expression, and inhibitors of TGF*β* signalling did not modulate PD-L1 expression [14].	mostly
colon cancer	ZEB and PD-L1 overexpression and a reduction in miR-200 were identified in budding areas in colon cancer tissues. PD-L1 overexpression led to low levels of miR-200a and miR-200c and high levels of ZEB1 and ZEB2 [15].	yes
oesophageal squamous cell carcinoma (OSCC)	TGF*β* induced ZEB1 and PD-L1 expression in epithelial-like OSCC cell lines. ZEB1 knockdown by RNA interference stimulated E-cadherin and suppressed PD-L1 mRNA and protein expression in a mesenchymal-like cell line. Because the promoter region of PD-L1 contains a binding site for ZEB1, the authors speculated that ZEB1 directly regulates PD-L1 expression, in addition to its indirect regulation through miR-200 [45].	mostly^a^
	PD-L1 expression promoted the mesenchymal phenotype in oesophageal cancer cells. Ablation of PD-L1 enhanced E-cadherin and suppressed several mesenchymal markers, and the opposite was observed upon PD-L1 overexpression [46].	yes
	TRAIL induced EMT in OSCC by upregulating PD-L1 expression. In addition, treatment with anti-PD-L1 antibodies inhibited TRAIL-mediated metastasis in mice [47].	yes
glioblastoma	In glioblastoma multi-forme cells, PD-L1 overexpression downregulated E-cadherin and upregulated N-cadherin and Vimentin, as well as the upstream transcription factors SLUG and *β*-catenin (but not ZEB1). Mechanistically, PD-L1 activated the EMT process via Ras binding and activating the downstream MEK/ERK signalling [48].	partly^a^
hepatocellular carcinoma	PD-L1 expression promoted EMT in sorafenib-resistant HepG2 SR and Huh7 SR cell lines via the PI3K/Akt pathway by activating SREBP-1 expression [49].	yes^a^
lung cancer	Tumour cell PD-L1 expression is regulated by the miR-200/ZEB1 axis in non-small cell lung cancer (NSCLC). Chen *et al.* postulate that this is caused by miR-200 binding sites on the mRNA of PD-L1. Induction of EMT using TGF*β* upregulated ZEB1 and PD-L1 expression, and constitutive ZEB1 expression enhanced PD-L1 expression on epithelial lung cancer cell lines. Conversely, PD-L1 was suppressed in mesenchymal cells upon ZEB1 knockdown or miR-200 overexpression [13].	yes
	Cytokine-driven EMT signalling reversibly upregulated PD-L1 expression in NSCLC cell line A549. Mechanistically, this was regulated by DNA methylation and NF-*κ*B signalling and required both 1 and TNF-*α* [50].	yes^a^
	There is a positive association between PD-L1 and phosphorylated Smad2 in NSCLC tumours, and 1 upregulated PD-L1 gene transcription in NSCLC in a Smad2-dependent manner [51].	yes^a^
	Knockdown of PD-L1 by small interfering RNAs (siRNAs) suppressed expression of mesenchymal markers and upregulated E-cadherin expression in H460 and H358 cells, and PD-L1 overexpression showed the opposite effect [52].	yes^a^
renal cell carcinoma	Downregulation of PD-L1 led to downregulation of the mesenchymal marker vimentin and upregulation of the epithelial marker E-cadherin, while PD-L1 upregulation enhanced vimentin and suppressed E-cadherin expression through activation of SREBP-1 [53].	yes^a^

^a^However, different mechanisms/pathways (also) appear to be involved.

Although findings of our model and experimental data are in good agreement, some results are partially conflicting: firstly, Noman *et al.* [14] reported that while PD-L1 expression in EMT-activated breast cancer cell lines is regulated via the SNAIL1/ZEB1/miR-200 axis, treatment of MCF7-2101 cells with TGF*β* or inhibition of TGF*β* in MCF sh-WISP2 cells did not modulate PD-L1 expression. However, the MCF7-2101 cell line already has a mesenchymal phenotype, evident from their vimentin expression and lack of E-cadherin; hence, additional TGF*β* is not expected to further increase PD-L1 expression by triggering EMT. Moreover, although the TGF*β* signalling inhibitors repressed SMAD2 activation in MCF7 sh-WISP2 cells, phosphorylated SMAD2/3 is only essential for SNAIL1 activation and not for the persistence of SNAIL1 expression [58]. Thus, the performed experiments cannot rule out that TGF*β* signalling modulates PD-L1 expression in these cell lines. Secondly, PD-L1 expression in certain cell lines is reportedly affected by silencing of ZEB1 but not of SNAIL1 [14,45]. Although SNAIL1 drives ZEB1 expression in the EMT core regulatory network model we used, this apparently contradictory finding can be reconciled by appreciating that the EMT transcriptional response is highly context specific [59,60]. Indeed, sustained expression of ZEB1 can be achieved by various EMT regulators and even by ZEB1 itself, resulting in an irreversible switch to the mesenchymal phenotype [39,61,62]. Thus, SNAIL1 silencing may be insufficient to affect PD-L1 in settings where ZEB1 is maintained via other means.

Even though partially conflicting findings between our model and published data can be reconciled, it is also likely that additional mechanisms by which PD-L1 and EMT mutually influence each other play a role depending on the studied cell line or cancer. For instance, PD-L1 expression can induce EMT by activating the TF SREBP-1c in hepatocellular and renal cell carcinoma [49,53], or by preventing SNAIL1 ubiquitination in triple-negative breast cancer [63]. In addition, ZEB1 can bind and silence IRF1, a TF of PD-L1 [64], and the activity of the CMTM family, which stabilizes PD-L1, correlates with EMT-TFs such as SLUG [65,66].

In the related mathematical model presented by Sahoo *et al.* [22], an indirect feedback mechanism of PD-L1-mediated E/cadherin inhibition was included. Results of their model agree qualitatively with those of our model in terms of the prediction that hybrid E/M cells are expected to be PD-L1 positive and hence immune-evasive. However, the predicted PD-L1 expression levels for the different EMT phenotypes differ substantially: Sahoo *et al.* [22] predict that the hybrid and mesenchymal phenotypes have almost equally high PD-L1 expression, whereas we predict that hybrid cells are closer to epithelial cells with respect to PD-L1 expression. They substantiated that PD-L1 expression increases with EMT scores by an analysis of gene expression in pan-cancer datasets and analysis of responses in several cell lines. For some of these data, intermediate EMT scores (interpreted as hybrid cells) have the tendency to only moderately increase the PD-L1 expression (e.g. figs 1E and F and 5E in [22]), which is more in line with our model predictions. A key difference in our respective approaches is that Sahoo *et al.* [22] employed RACIPE (random circuit perturbation) [67], which yields semi-quantitative predictions based on network topology, whereas we used a mechanistic approach based on appropriate miRNA–mRNA dynamics. As a consequence, only the inhibition of miR-200 on PD-L1 is present in the model by Sahoo *et al.* [22], because the inhibition of PD-L1 by miR-200, which arises as a consequence of miRNA–mRNA dynamics, cannot be captured by using the Hill-type dynamics in RACIPE. Nevertheless, the experimental work is required to investigate whether miR-200 degradation is enhanced upon binding to PD-L1. Moreover, the involved mechanisms and factors affecting PD-L1 expression and EMT are probably context and cell-line specific, and further research will be required to unravel this complicated interplay between factors. Our model forms a basis to include such factors.

A limitation of our model is its relatively crude description of IFN*γ*-induced PD-L1 expression. To keep the model simple, we used a steady-state approximation of the simplified model by Quaiser *et al.* [25], which only describes the first 15 min of JAK–STAT signalling before transcriptional feedback occurs. Nevertheless, our model’s predicted IRF1 temporal dynamics roughly agree with the JAK–STAT model by Rateitschak *et al.* (cf. fig. 4A in[37]). Comparison of these results with those of a highly simplified IFN*γ*-driven PD-L1 expression model showed that the role of JAK–STAT signalling primarily creates a ‘switchlike’ response to IFN*γ* (cf. figure 2*b* and electronic supplementary material, figure S4*b*).

The predicted decrease of SNAIL1 levels required for EMT in the presence of IFN*γ* (as indicated by the leftward shift in the bifurcation diagrams in figure 2*c*,*d*) is noteworthy in itself; although several studies report on the modulation of PD-L1 expression as a result of EMP, the mechanism by which PD-L1 could modulate EMT remains unclear [12]. Here, we show that the additional degradation of the PD-L1–miR-200 complex in the presence of IFN*γ* could be affecting miR-200 levels sufficiently to affect EMT, without the need for additional mechanisms.

Another interesting result is the acceleration of EMT by IFN*γ* as a result of the increased interaction between miR-200 and PD-L1. This is reminiscent of recent theoretical work that showed that intermediate EMT states or unobservable microstates might accelerate the EMT process [68,69], i.e. an increased PD-L1 level could be viewed as such a microstate. (Single-cell) transcriptomics experiments with sufficient time resolution would help decipher the different involved mechanisms and how they influence the EMT temporal dynamics.

The results reported here may have diagnostic and therapeutic implications. PD-L1 has been proposed as a biomarker to predict the efficacy of PD-1/PD-L1 blockade therapy [70], yet the multi-factorial mechanisms underlying PD-L1 membrane expression complicate its use as an exclusive biomarker [71,72]. For example, oncogene-driven PD-L1 expression caused by EMT is constitutive, diffuse and distinct from inflammation-driven PD-L1 expression, which may occur more focally and during a limited time window. The latter is often associated with the presence of an immune infiltrate, while the former is associated with a lack thereof [71], and it has been proposed that this combination of PD-L1 expression and the presence of immune infiltrate affects the response to PD-1/PD-L1 blockade therapy [73]. Our findings highlight how oncogenic and inflammation-driven PD-L1 expression might interact and give rise to strongly increased PD-L1 levels. PD-L1 on tumour cells is an important contributor to immune evasion through inhibition of CD8+ T cell cytotoxicity [74]. Consistently, in metastatic urothelial cancer, lack of response to anti-PD-L1 treatment occurred particularly in patients with tumours that showed exclusion of CD8+ T cells [75]. Combined treatment with TGF*β*-blocking and anti-PD-L1 antibodies invoked anti-tumour immunity and tumour regression by facilitating T cell infiltration, which could be due to EMT-independent TGF*β* signalling [76], but possibly also via the EMT-dependent path we have presented here. Another opportunity could be the use of therapeutic siRNA [77]: in contrast to anti-PD-L1 antibodies, siRNA affects PD-L1 mRNA, which, according to our model, should free up miR-200 for its anti-EMT effect, a prediction that is supported by (partial) EMT reversal following downregulation of PD-L1 using siRNA/shRNA [40,52].

In conclusion, we developed a mathematical model that describes the crosstalk between IFN*γ*-induced PD-L1 and EMT, which is in good agreement with experimental findings. In addition, our model sheds light on potential mechanisms behind EMT-mediated immune evasion, and primary, adaptive, or acquired resistance to immunotherapy. Our (simplified) model can serve as a starting point to explore additional EMP and immune crosstalk mechanisms. In particular, we propose embedding the presented model in a multi-scale model to explicitly describe the local effects of the adaptive immune response and the effects of TGF*β* on the tumour microenvironment. Improved understanding of the interaction between the immune response and EMP is indispensable for developing better diagnostic and therapeutic options for cancer patients.

## 4. Material and methods

### 4.1. Simplified ternary chimera switch model

The simplified TCS model [26] is built on the theoretical framework for microRNA-TF chimera toggle-switches defined in the study by Lu *et al.* [28]. See electronic supplementary material, Methodsfor the model definition, theoretical framework, and used parameters.

### 4.2. IFN*γ*-PD-L1 model

The IFN*γ*–PD-L1 model consists of two parts: (IFN*γ*–)JAK–STAT signalling, for which we use a steady-state approximation of an existing model, and STAT–PD-L1 regulation which we developed following the theoretical framework underpinning the simplified TCS model. We discuss these parts in more detail below.

#### 4.2.1. (IFN*γ*–)JAK–STAT

For the IFN*γ*–PD-L1 model, we use the JAK–STAT model presented in [25] (see electronic supplementary material, Methods). The dynamics of IFN*γ*–STAT and STAT–PD-L1 are well separated in time (figure 1*c*,*d*), with JAK–STAT dynamics happening on a time scale of minutes [34] and STAT–PD-L1 dynamics happening on a time scale of hours [35,36]. Therefore, we simplified our IFN*γ*–PD-L1 model by assuming the JAK–STAT model is in aquasi-steady state; we fitted its steady state by a Gompertz function (see electronic supplementary material, figure S8) and inserted this approximate relationship between IFN*γ* and STAT in our STAT–PD-L1 model.

#### 4.2.2. STAT–PD-L1

The STAT–PD-L1 submodel (see also electronic supplementary material, figure S1A) consists of the following ordinary differential equations (ODEs):

4.1 $$\mathrm{IRF}1\;\mathrm{mRNA}\;:\;\;\frac{{\text{dm}}_{\text{F}}}{\text{dt}}={\text{g}}_{m_{\text{F}}}{\text{H}}^{\text{S}}\left(\text{STAT},\;\lambda_{\text{STAT},{\text{m}}_{\text{F}}}\right)-{\text{k}}_{{\text{m}}_{\text{F}}}{\text{m}}_{\text{F}},$$

4.2 $$\text{IRF1 protein}:\;\frac{\text{dF}}{\text{dt}}={\text{g}}_{\text{F}}{\text{m}}_{\text{F}}-{\text{k}}_{\text{F}}\text{F},$$

4.3 $$\text{PD-L1 mRNA}:\;\frac{{\text{dm}}_{\text{P}}}{\text{dt}}={\text{g}}_{{\text{m}}_{\text{P}}}{\text{H}}^{\text{S}}(\text{F},\;\lambda_{\text{F},{\text{m}}_{\text{P}}})-{\text{k}}_{{\text{m}}_{\text{P}}}{\text{m}}_{\text{P}},$$

4.4 $$\text{PD-L1}\;\text{in}\;\text{ER}:\;\frac{{\text{dP}}_{\text{ER}}}{\text{dt}}={\text{g}}_{{\text{P}}_{\text{ER}}}{\text{m}}_{\text{P}}-{\text{k}}_{\text{ER},\text{G}}{\text{P}}_{\text{ER}},$$

4.5 $$\mathrm{PD}\text{-}L1\;\mathrm{in}\;\mathrm{Golgi}\;:\;\;\frac{dP_{G}}{dt}=k_{\mathrm{ER},G}P_{\mathrm{ER}}-k_{G,M}P_{G}$$

4.6 $$\mathrm{and}\;\;\;\;\;\mathrm{PD}\text{-}L1\;\mathrm{membr}.\;:\;\;\frac{dP_{M}}{dt}=k_{G,M}P_{G}-{\text{k}}_{{\text{P}}_{\text{M}}}P_{M}.$$

This model uses the abundance of STAT (in molecules) as input and includes IRF1 and PD-L1 using appropriate TF-TF dynamics (*H*^*S*^) from the theoretical framework by Lu *et al.* [28] (see electronic supplementary material, Methods). Transport of PD-L1 through various cellular compartments is modelled with a constant rate from compartment to compartment, with rates assumed to be similar to Lippincott-Schwartz *et al.* [78]. Model parameters are provided in table 2; some parameters were considered to be similar to those of the simplified TCS model.

**Table 2.** Variables and parameters used for the STAT–PD-L1 model. The top panel shows variable names and production and degradation rates; the bottom panel shows parameters for the shifted Hill functions of the interactions. Degradation rates *k* are in h^−1^, production rates *g* in molecules h^−1^ and thresholds $B_{A}^{0}$ in molecules.

		prod. rate *g*		degr. rate *k*	
IRF1 mRNA	*m*_*F*_	$g_{m_{F}}$	30	$k_{m_{F}}$	0.5
IRF1 protein	*F*	*g*_*F*_	100	*k*_*F*_	0.1
PD-L1 mRNA	*m*_*P*_	$g_{m_{P}}$	30	$k_{m_{P}}$	0.5
PD-L1 in ER	$P_{ER}$	$g_{P_{ER}}$	100	$k_{ER,G}$	1.68
PD-L1 in Golgi complex	*P*_*G*_			*k*_*G*,*M*_	1.8
PD-L1 on membrane	*P*_*M*_			$k_{P_{M}}$	0.15

	threshold $B_{A}^{0}$		Hill coefficient $n_{BA}$		max. fold change $\lambda_{BA}$	
Act. *m*_*F*_ by STAT	${STAT}_{m_{F}}^{0}$	2 × 10^6^	$n_{STAT,m_{F}}$	10	$\lambda_{STAT,m_{F}}$	10
Act. *m*_*P*_ by *F*	$F_{m_{P}}^{0}$	10^5^	$n_{F,m_{P}}$	3	$\lambda_{F,m_{P}}$	10

### 4.3. Combined model

To create our combined model, we connected the IFN*γ*-induced JAK–STAT signalling model and simplified TCS model to the central STAT–PD-L1 model (see figure 1*b*). In this combined model, equations (4.7) to (4.9) are used instead of equations (4.3), (4.4) and electronic supplementary material, (S7) (see electronic supplementary material, Methods) to include the interaction of miR-200 with PD-L1. Here, $Y_{\mu ,m_{Z}}(\mu )$ in the updated miR-200 equation (equation (4.9)) corresponds to *Y*_*μ*_(*μ*) in the simplified TCS model (electronic supplementary material, equation (S7)).

4.7 $$\mathrm{PD}\text{-}L1\;\mathrm{mRNA}\;:\;\frac{dm_{P}}{dt}=g_{m_{P}}H^{S}(F,\lambda_{F,m_{P}})-m_{P}Y_{m}(\mu )-k_{m_{P}}m_{P},$$

4.8 $$\mathrm{PD}\text{-}L1\;\mathrm{in}\;\mathrm{ER}\;:\;\frac{dP_{ER}}{dt}=g_{P_{ER}}m_{P}L(\mu )-k_{ER,G}P_{ER}$$

4.9 $$\mathrm{and}\;\mathrm{miR}\text{-}200\;:\;\frac{d\mu }{dt}=g_{\mu }H^{S}(Z,\lambda_{Z,\mu })H^{S}(S,\lambda_{S,\mu })-m_{Z}Y_{\mu ,m_{Z}}(\mu )-m_{P}Y_{\mu ,m_{P}}(\mu )-k_{\mu }\mu .$$

The connection between miR-200 and PD-L1 is done using appropriate miRNA–mRNA dynamics (*L* and *Y* functions) from the theoretical framework by Lu *et al.* [28] (see electronic supplementary material, Methods). To accommodate a low number of binding sites for miR-200 on the mRNA of PD-L1 (i.e. two binding sites according to Chen *et al.* [13]), we adapted the parameters for *L*(*μ*), *Y*_*m*_(*μ*) and $Y_{\mu ,m_{P}}(\mu )$ from electronic supplementary material, table S1 (*n* = 6) to the values as shown in table 3 (*n* = 2). Note that the inhibition of miR-200 on PD-L1 automatically leads to inhibition of PD-L1 on miR-200 as the included miRNA–mRNA dynamics [28] result in additional miR-200 degradation ($m_{P}Y_{\mu ,m_{P}}(\mu )$ in equation (4.9); see electronic supplementary material, figure S6). We approximately matched the results of our combined model in the absence of IFN*γ* with the results of the TCS model by compensating for this additional degradation of miR-200. We achieved this by decreasing the basal degradation rate of miR-200 from $k_{\mu }=0.05$ (electronic supplementary material, table S2) to $k_{\mu }=0.0475$ (see electronic supplementary material, figure S2). We considered *μ*_0_ = 10^4^ molecules, following Jolly *et al.* [26].

**Table 3.** Rates used in the *L*(*μ*), *Y*_*m*_(*μ*) and $Y_{\mu ,m_{P}}(\mu )$ functions in the combined model for *n* = 2.

*n* (no. of miRNA binding sites)	0	1	2
*l*_*i*_ [h^−1^]	1	0.3	0.05
*γ*_*mi*_ [h^−1^]	0	0.2	1
*γ*_*μi*_ [h^−1^]	0	0.05	0.5

In addition, since the JAK–STAT model has [STAT1p_2] as output in nM we need to convert to number of molecules to use STAT1p_2 as input in the STAT–PD-L1 model. As in the study by Jolly *et al.* [26], we use a cell volume of 10 000 μm^3^, such that 1 nM amounts to roughly 6020 molecules (6.02 × 10^23^ · 10^−9^ · 10 000 × (10^−5^)^3^). Note that this cell volume is on the high side, as typical animal cells are 10–20 μm in diameter (approx. 500–4000 μm^3^) [79, p. 529]). However, because of our IFN*γ*–STAT steady-state approximation, a decrease in cell volume by a factor 10 can be compensated by multiplying the inducing IFN*γ* signal also by a factor 10 to yield the same amount of STAT, and hence, the same model result. Table 4 shows all model components and their units in our combined model.

**Table 4.** List of regulators in the combined model.

regulator	symbol	units
^a^extra-cellular IFN*γ*	[*IFNγ*]	nM
[STAT1p_2]	*x*_10_	nM
STAT1p_2	*STAT*	no. molecules
IRF1 mRNA	*m*_*F*_	no. molecules
IRF1 protein	*F*	no. molecules
PD-L1 mRNA	*m*_*P*_	no. molecules
PD-L1 in endoplasmatic reticulum	*P_ER_*	no. molecules
PD-L1 in Golgi complex	*P*_*G*_	no. molecules
PD-L1 on cell membrane	*P*_*M*_	no. molecules
miR-200	*μ*	no. molecules
ZEB1 mRNA	*m*_*Z*_	no. molecules
ZEB1 protein	*Z*	no. molecules
^a^SNAIL1 protein	*S*	no. molecules

^a^Model inputs.

### 4.4. Simulation and analysis

For model simulations, we used COPASI (complex pathway simulator) (RRID:SCR_014260) [80], and model files are included in the electronic supplementary material [81].

Analysis in R (R Project for Statistical Computing, RRID:SCR_001905) [82] was performed with RStudio (RStudio, RRID:SCR_000432) [83] and the tidyverse [84] packages.

## Data Availability

COPASI models supporting this article have been uploaded as part of the electronic supplementary material [81]. Data and code to generate the article figures are available at https://doi.org/10.5281/zenodo.7034918 (G. Burger *et al.* 2022) [85].
